# Medicinal plants used in managing diseases of the respiratory system among the Luo community: an appraisal of Kisumu East Sub-County, Kenya

**DOI:** 10.1186/s13020-020-00374-2

**Published:** 2020-09-03

**Authors:** James Kiamba Mailu, Joseph Mwanzia Nguta, James Mucunu Mbaria, Mitchel Otieno Okumu

**Affiliations:** 1 Department of Public Health, Pharmacology, and Toxicology, Faculty of Veterinary Medicine, University of Nairobi, P.O Box 29053-00625, Nairobi, Kenya; 2 Department of Pharmacy, Kenya Medical Training College, Kisumu Campus Kenya, P.O Box 1594, Kisumu, Kenya; 3 Department of Pharmacy, Jaramogi Oginga Odinga Teaching and Referral Hospital, P.O Box 849-40100, Kisumu, Kenya

**Keywords:** Ethnopharmacology, Medicinal plants, Kisumu East, Luo, Ethnomedicinal, Ethnobotanical, Respiratory diseases, Cough

## Abstract

**Background:**

Poor access to healthcare in rural communities causes many people to seek herbalists who use medicinal plants for the treatment of various disease conditions. Most knowledge of traditional herbal medicine makes use of indigenous remedies which are often undocumented and are at risk of being lost. The preservation of this knowledge may facilitate scientific inquiry into promising new therapeutic molecules.

**Methods:**

Semi-structured questionnaires were used to collect the sociodemographic information of 30 herbalists in Kisumu East Sub County. The local names of medicinal plants used in managing illnesses of the respiratory system, their habit, active parts, indications, methods of preparation, routes of administration, scientific identity, and conservation status were also recorded. Other reported traditional uses, pharmacological activities, and toxicological data were identified via a literature search.

**Results:**

Most herbalists were female (86.7%), aged between 61 and 70 years (43.3%) with no formal education (56.7%), and had 21–30 years of practice (30%). 44 plant species, belonging to 43 genera and 28 families were identified. Leguminosae and Rutaceae plant families were predominant, leaves were frequently used (33%), and trees were the most common habit (44.4%). Most plants were collected in the wild (79.2%), preparation was mainly by decoction (68.8%), and the administration was mainly orally. The main indication was cough and 79.5% of all documented plant species had previously been reported to have a pharmacological activity relevant to the mitigation of respiratory illnesses. Toxicological data was available for 84.1% of the plant species identified.

**Conclusions:**

The predominant use of roots, root barks, and root tubers by herbalists in Kisumu East Sub County threatens to negatively impact the ecological survival of some plant species. The preservation of herbalists’ knowledge of medicinal plants in the study area is a pressing concern considering their advanced age and little formal education. There is a need to conserve some of the medicinal plants documented in this study. The medicinal claims made by herbalists also warrant scientific scrutiny.

## Background

The global burden of respiratory diseases makes for daunting reading. Lower respiratory tract infections (LRTI) and chronic obstructive pulmonary disease (COPD) reportedly claimed 6 million human lives in 2016 [1]. The prevalence of COPD in Sub Saharan Africa has been reported to be between 4 and 25% and > 100,000 deaths have been linked to non-communicable diseases including those of the respiratory system [2, 3]. Diseases of the respiratory system hurt individual productivity and are responsible for more than 10% of all disability-adjusted life years [4].

According to a 2013 Kenya National Bureau of Statistics (KNBS) economic survey, pneumonia, and tuberculosis were responsible for 13.7% of all total deaths in the Nyanza region [5]. It is important to note that illnesses of the upper respiratory tract are the second leading cause of death in Kisumu County [6]. Poor access to healthcare and scarcity of health resources in rural areas such as many parts of Kisumu East Sub County causes many inhabitants of such areas to rely on indigenous plant resources to manage common diseases including those that affect the respiratory system. Plant-based indigenous remedies may be key in the future management of respiratory system diseases [7]. However, the potential of this resource is largely untapped due to inadequate documentation by the herbalists who prepare the remedies.

The rapid development of infrastructure in Sub Saharan Africa including Kenya threatens to destroy cultural lands where medicinal plants are cultivated. This is problematic given that the knowledge of these plant resources is mostly an extension of people’s culture [8, 9]. Herbalists are usually the custodians of medicinal plants in these communities. By documenting the knowledge held by herbalists, vital information on the medicinal plants may be preserved. The current study aimed to collect ethnobotanical data on medicinal plants used by herbalists in the management of respiratory diseases in Kisumu East Sub County.

## Materials and methods

### Ethical approval and consent to participate in the study

Ethical approval for the study was obtained from the Biosafety, Animal Use and Ethics committee of the University of Nairobi (Ref: FVM BAUEC/2019/210). Approval was additionally sought from regional administrators (the area chief and assistant chief) who were duly notified of the study’s objectives. The scope, possible benefits, and risks of the study were explained to willing participants (herbalists) and consent forms were made available to them for signing.

### Study area

**Fig. 1:**
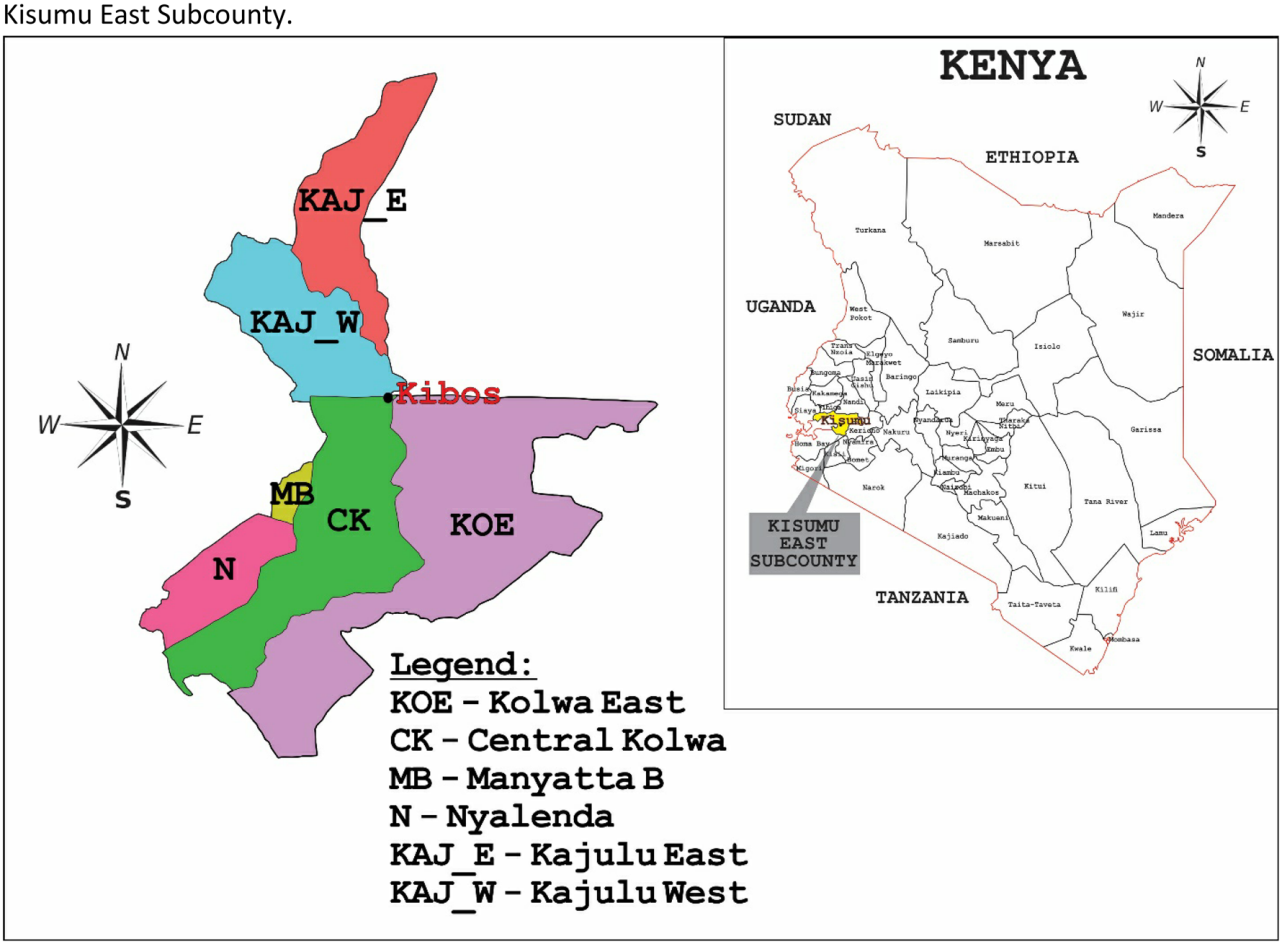
Map of Kenya showing Kisumu County and Kisumu East Sub County

The study was conducted in Kisumu East Sub County in Western Kenya (Fig. 1). The study area is approximately 365 km from Nairobi (the administrative capital of Kenya) and covers an area of approximately 135 km^2^. It lies within latitudes 0° 20′ South and 0° 50′ South and longitudes 33° 20′ E and 35° 20′ E and comprises of several administrative wards including Kolwa Central, Kolwa East, Manyatta B, Nyalenda A, and Kajulu East and West [10]. Moreover, the population in this area is about 220,977 according to the 2019 Kenya Population and Housing Census [5]. It receives an annual relief rainfall of between 1200 and 1300 mm and annual temperatures range between 20 and 35 °C. The major economic activities of residents include fish farming, and agriculture (sugar, livestock, and poultry farming) [10].

### Data collection

The study was conducted between March and September 2019. Ethnobotanical data were obtained by using semi-structured questionnaires. The target respondents were local herbalists with good ethnobotanical knowledge of the plants used in managing respiratory diseases and related symptoms. Thirty local herbalists were selected for interviews which were conducted both in Kiswahili and Luo dialect with the aid of a botanist familiar with the languages. Each of the respondents was interviewed individually to ensure confidentiality. The interviews sought to answer the following questions;

- Which plant parts are most commonly used in preparing the indigenous remedies indicated for respiratory illnesses?
- Which methods are adopted in preparing the indigenous remedies?
- Which respiratory illnesses are most commonly treated with medicinal plants in the study area?
- Which plant species are used in the preparation of the remedies?
- How are the indigenous remedies administered? See Additional file 1.

### Collection and identification of plant specimens

Several trips were made to the homesteads of the herbalists where voucher specimens were collected and pressed and later identified by a botanist before being deposited at the University of Nairobi Herbarium. Information on the vernacular name, plant part used, plant habit (i.e. the general appearance, growth form, or architecture), plant status, method of preparation, and route of administration were collected.

### Literature search strategy

A literature search was conducted on MEDLINE, PubMed, PubMed Central (PMC), Google Scholar, the Directory of Open Access Journals (DOAJ), The Journal Author Name Estimator (JANE), University repositories, and from grey literature to identify relevant articles/theses/ reference material containing information on previously reported traditional uses, pharmacological/chemical activities, and toxicological data on the medicinal plants indicated for the management of respiratory illnesses in Kisumu East Sub County. Studies were excluded if they were not in English.

### Data analysis

Frequencies and percentages were used to analyze the sociodemographic data of the herbalists. The relative frequency of citation (RFC) was used to evaluate the ethnobotanical data.

### Relative frequency of citation (RFC)

This was done to determine the number of herbalists who considered particular plant species were worth mentioning in the management of diseases of the respiratory system. The value was calculated using the formula described by Tardio and Santayana [11];

$${\text{RFCs}} = \frac{FCs}{N} = \mathop \sum \limits_{i = i1}^{iN} URi/N$$

where Fc is the number of herbalists who cited a particular species and N is the total number of herbalists (Table 1).

**Table 1:** Demographic characteristics of herbalists interviewed in Kisumu East Sub County (n = 30) during the study period

Variable (n = 30)	Frequency (percentage)
Gender	
Male	4 (13.3)
Female	26 (86.7)
Age	
31–40	5 (16.7)
41–50	1 (3.3)
51–60	5 (16.7)
61–70	13 (43.3)
> 70	6 (20)
Education	
None	17 (56.7)
Basic	12 (40)
Secondary	1 (3.3)
Years of experience	
1–10	5 (16.7)
11–20	8 (26.7)
21–30	9 (30)
31–40	4 (13.3)
41–50	2 (6.7)
> 50	2 (6.7)

## Results

### Socio-demographic characteristics of the herbalists who were interviewed

86.7% of all herbalists were female, and aged between 61 and 70 years of age (43.3%) (Table 1). The average age of the female herbalist was 61.6 years while the average age of their male counterparts was 51.5 years of age. Seventeen of the herbalists (56.7%) had no formal education while only 1 had secondary education (Table 1). It was observed that both male and female herbalists had extensive years of practice. The mean years of practice for male and female herbalists in the study area were 27 years and 25 years for male and female herbalists respectively.

### Diversity of medicinal plants identified and their use

**Fig. 2:**
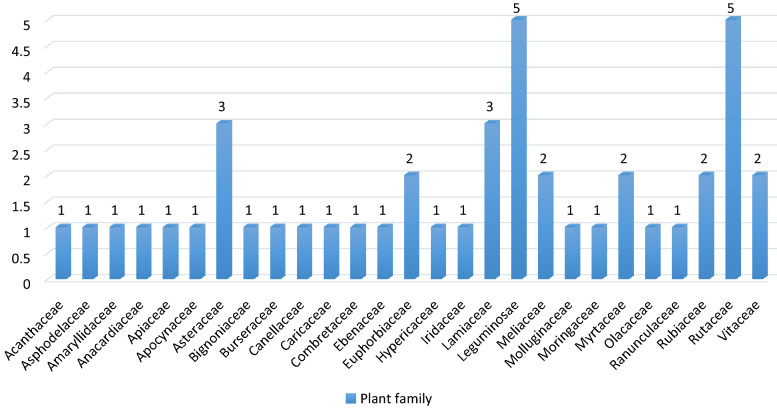
Plant families used by herbalists in Kisumu East Sub County to manage diseases of the respiratory system

Table 2 is a summary of the family, scientific name, local name, voucher number, habit, status, and the part used, indication, method of preparation, route of administration and relative frequency of citation of medicinal plants used in managing respiratory diseases by herbalists in Kisumu East Sub County. Forty-four plant species belonging to 43 genera distributed among 28 families were reportedly used in herbal preparations for the management of respiratory infections (Table 2). Leguminosae and Rutaceae families predominated with 5 species each, followed by Asteraceae and Lamiaceae families with 3 species each (Fig. 2). Euphorbiaceae, Meliaceae, Myrtaceae, Rubiaceae, and Vitaceae family had 2 species each (Fig. 2). The other families had 1 species only. The identified 44 species comprised of trees (44.4%), shrubs (37.8%), herbs (8.9%), climbers (6.7%), and corms (2.2%) (Table 2). A majority of the plants were sourced from the wild (79.2%) while some were grown in the homestead (20.8%). The most cited plants were *Euclea divinorum, Tylosema fassoglensis, Carissa edulis, Harrisonia abyssinica, Zanthoxylum gilletii,* and *Warburgia salutaris* with RFC values of 0.73, 0.67, 0.67, 0.6, 0.5, 0.47 and 0.47 respectively (Table 2).

**Table 2:** Plants used in managing diseases of the respiratory system among the Luo community of Kisumu East Sub County

Family	Scientific name	Local name	Voucher no.	Habit	Status	Part used	Condition managed	Mode of preparation	Route of administration	RFC
Acanthaceae	*Acanthus polystachyus* Delile	Not provided	JM2019/284/003	Shrub	Wild	Roots	Cough	Decoction	Oral	0.07
Asphodelaceae	*Aloe kedongensis* Reynolds	Ogaka	JM2019/194/030	Shrub	Wild	Leaves	Asthma, Pneumonia	Concoction	Oral	0.23
Amaryllidaceae	*Allium sativum* L.	Otungu	JM2019/194/031	Herb	Cultivated	Bulb	Allergies	Chewing or as a concoction	Oral	0.03
Anacardiaceae	*Rhus natalensis* Bernh.	Sagla	JM2019/194/021	Shrub	Wild	Roots	Asthma	Concoction	Oral	0.07
Apiaceae	*Steganotaenia araliacea* Hochst.	Nyaniang-liech	JM2019/118/006	Tree	Wild	Roots or stem bark	Pneumonia	Decoction	Oral	0.03
Apocynaceae	*Carissa edulis* (Forssk.) Vahl	Ochuoga	JM2019/194/022	Shrub	Wild	Roots	Common cold, pneumonia, asthma	Decoction	Oral	0.67
Asteraceae	*Artemisia annua* L.	Nyumba	JM2019/269/001	Herb	Wild or cultivated	Leaves	Asthma	Decoction	Oral	0.03
	*Microglossa pyrifolia* (Lam.) Kuntze	Nyabung-odide	JM2019/194/006	Shrub	Wild	Leaves or roots	Cough	Maceration or as a concoction	Oral	0.07
	*Tithonia diversifolia* (Hemsl.) A. Gray	Mafua/maua	JM2019/194/012	Shrub	Wild	Stem bark or leaves	Asthma	Concoction	Oral	0.03
Bignoniaceae	*Kigelia africana* (Lam.) Benth.	Yago	JM2019/194/003	Tree	Wild or cultivated	Fruit or stem bark	Pneumonia	Decoction	Oral	0.3
Burseraceae	*Commiphora africana* (A.Rich.) Engl.	Arupiny	JM2019/194/007	Tree	Wild	Roots	Pneumonia	Decoction	Oral	0.17
Canellaceae	*Warburgia salutaris* (G.Bertol) Chiov	Abaki	JM2019/244/001	Tree	Wild or cultivated	Stem bark	Asthma, allergy, chest pain, pneumonia	Decoction	Oral	0.47
Caricaceae	*Carica papaya* L.	Apoyo	JM2019/269/002	Tree	Cultivated	Roots or leaves	Bronchitis	Decoction	Oral	0.07
Combretaceae	*Terminalia brownii* Fresen	Minera/Manera	JM2019/058/016	Tree	Wild or cultivated	Stem bark	Asthma, pneumonia, common cold	Decoction	Oral	0.2
Convolvulaceae	*Ipomoea kituiensis* Var	Obinju	JM2019/194/028	Shrub	Wild	Leaves	Cough	Decoction	Oral	0.03
Ebenaceae	*Euclea divinorum *Hiern.	Ochol	JM2019/194/023	Shrub	Wild	Roots	Pneumonia, asthma	Decoction	Oral	0.73
Euphorbiaceae	*Croton megalocarpus* Del.	Ofunja muri	JM2019/194/015	Tree	Wild	Leaves	Pneumonia	Decoction	Oral	0.17
	*Croton dichogamous* Pax	Rachar	JM2019/178/001	Tree	Wild	Roots	Asthma	Decoction	Oral	0.1
Hypericaceae	*Harungana madagascariensis* Lam. Ex Poir	Aremo	JM2019/058/005	Tree	Wild	Leaves	Cough	Decoction	Oral	0.2
Iridaceae	*Gladiolus dalenii* Van Geel	Obuya	JM2019/284/001	Corm	Wild	Corm	Asthma, allergy	Powdered	Inhalation	0.1
Lamiaceae	*Clerodendrum myricoides* (Hochst.) R.Br.ex Vatke	Okwerogweno/sangla	JM2019/058/021	Shrub	Wild	Roots or leaves	Pneumonia, asthma	Decoction	Oral	0.17
	*Plectranthus barbatus* Andr.	Okita	JM2019/058/009	Shrub	Wild	Leaves	Asthma, pneumonia, allergy	Decoction	Oral	0.33
	*Vitex doniana* Sweet	Kalemba	JM2019/194/009	Tree	Wild	Leaves or stem bark	Allergies, common cold	Decoction	Oral	0.03
Leguminosae	*Acacia robusta* Burch.	Otiep	JM2019/214/001	Tree	Wild	Stem bark or root bark	Bronchial obstruction	Concoction	Oral	0.03
	*Albizia zygia* (DC.) J.J.Macbr.	Oturbam	JM2019/224/002	Tree	Wild	Stem bark	Pneumonia	Decoction	Oral	0.1
	*Rhynchosia elegans* var. elegans	Jandarusi/Jandalusi	JM2019/284/002	Herb	Wild	Root tubers	Cough	Concoction	Oral	0.03
	*Tamarindus indica* L.	Chwaa	JM2019/194/018	Tree	Wild or cultivated	Fruit or stem bark	Cough, general body malaise	Decoction	Oral	0.03
	*Tylosema fassoglense* (Kotschy ex Schweinf.) Torre & Hillc.	Ombasa	JM2019/194/016	Climber	Wild	Roots	Flu, pneumonia, asthma	Decoction	Oral	0.67
Meliaceae	*Azadirachta indica* (L) Burm.	Mwarubaine	JM2019/269/003	Tree	Wild or cultivated	Leaves	Cough	Decoction	Oral	0.3
	*Khaya senegalensis* Desr. A. Juss	Tido	JM2019/194/019	Tree	Wild	Stem bark	Common cold, cough	Decoction	Oral	0.47
Molluginaceae	*Mollugo nudicaulis* Lam.	Ataro	JM2019/138/001	Herb	Wild	Leaves	Cough	Chewed or as a decoction	Oral	0.03
Moringaceae	*Moringa oleifera* Lam.		JM2019/269/004	Tree	Cultivated	Leaves	General body malaise	Decoction	Oral	0.13
Myrtaceae	*Eucalptus camaldulensis* Dehnh	Bao	JM2019/269/005	Tree	Wild or cultivated	Leaves	Common cold	Decoction	Oral	0.33
	*Syzygium cumini* (L.) Skeels.	Jamna	JM2019/194/008	Shrub	Wild	Stem bark	Cough	Concoction	Oral	0.03
Olacaceae	*Ximenia americana* L.	Olemo	JM2019/269/006	Shrub	Wild	Roots or stem bark	Cough	Concoction	Oral	0.07
Ranunculaceae	*Clematis hirsuta* Guill. & Perr	Achogo	JM2019/269/007	Climber	Wild	Leaves	Common cold	Decoction	Oral	0.1
Rubiaceae	*Gardenia ternifolia* Schumach. & Thonn.	Rayudhi	JM2019/194/014	Shrub	Wild	Roots	Cough, Pneumonia	Decoction	Oral	0.13
	*Keetia gueinzii* (Sond.) Bridson	Atego	JM2019/264/001	Shrub	Wild	Root bark	Asthma, pneumonia, coughing, allergy	Powdered	Inhalation	0.2
Rutaceae	*Harrisonia abyssinica* Oliv.	Pedo	JM2019/194/001	Shrub	Wild	Roots	Cough, pneumonia, asthma	Decoction	Oral	0.6
	*Teclea nobilis* Del.	Madat midat	JM2019/194/024	Tree	Wild	Roots or leaves	Asthma, common cold	Decoction	Oral	0.2
	*Toddalia asiatica* L.	Ajua Nyalwet-kwach	JM2019/194/017	Shrub	Wild	Leaves or roots	Common cold, pneumonia, throat infection	Concoction	Oral	0.33
	*Zanthoxylum chalybeum* (Eng) Engl.	Roko	JM2019/269/008	Tree	Wild	Stem bark or root bark	Pneumonia	Decoction	Oral	0.03
	*Zanthoxylum gilletii* (De Wild.) P.G Waterman	Sogo-maitha	JM2019/224/001	Tree	Wild or cultivated	Stem bark	Asthma, pneumonia, coughing, General body malaise	Decoction	Oral	0.5
Vitaceae	*Cissus rotundifolia* (Forssk.) Vahl	Minya/katera	JM2019/194/026	Climber	Wild	Leaves	Throat infection, pneumonia, coughing	Decoction	Oral	0.2
	*Rhoicissus revoilii* Planch	Rabong’o	JM2019/269/009	Shrub	Wild	Root tubers	General body malaise	Decoction	Oral	0.17

*RFC* Relative frequency of citation

**Fig. 3:**
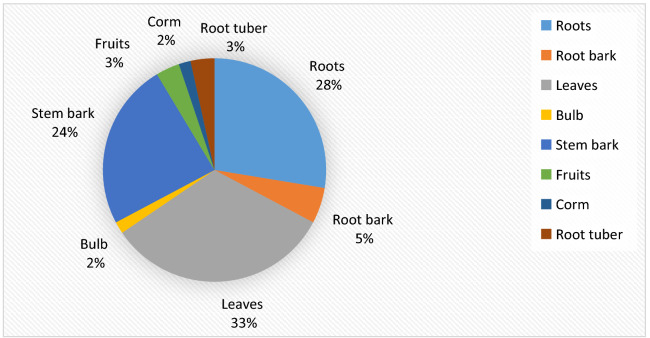
Plant parts used by herbalists in Kisumu East Sub County to manage diseases of the respiratory system

The different plant parts used by herbalists to manage respiratory illnesses in Kisumu East Sub County are summarized in Fig. 3. Leaves were the most frequently used parts (33%), followed by roots (28%) and stem bark (24%). Root bark, fruits, corms, bulbs, and root tubers accounted for 15%. Roots, root bark, root tuber, and stem bark accounted for 60% of plant parts used in the management of diseases of the respiratory system (Fig. 3).

### Dosage, mode of preparation, and route of administration

Various methods were used to prepare herbal medicine used for managing diseases of the respiratory system in the study area (Table 2). The most common method was decoction (68.8%), concoction (20.8%), and chewing (4.2%) (Table 2). Other methods of preparation included cold maceration, powdering, and crushing before instillation in the nostrils which accounted for 2.1% respectively (Table 2). The main route of administration of the indigenous remedies prepared by the traditional medicine practitioners was oral (Table 2).

### Pharmacological and toxicological reports on the medicinal plants documented in this study

Of the 44 plant species documented in this study, 95.5% had studies that had reported their pharmacological/chemical activity (Table 3). Moreover, 79.5% (35/44) of the documented medicinal plants had previously been reported to be effective against microorganisms that are associated with respiratory illnesses and 84.1% of the plant species had toxicological data (Table 3).

**Table 3:** Previously reported traditional uses, documented pharmacological/chemical activity, and toxicological data on the medicinal plants indicated for managing diseases of the respiratory system by herbalists in Kisumu East Sub County

Plant name	Previously reported traditional use	Reported pharmacological/chemical activity	Type of study	Toxicological data
*Acanthus polystachyus* Delile	Malaria [12], scorpion bite [13]	Antimalarial activity [14]	In vivo (Swiss albino mice) [14]	The methanol leaf extract was reported to be non-toxic in mice with a median lethal dose of > 2000 mg/kg [14]
*Aloe kedongensis* Reynolds	Malaria [15]	Antiplasmodial activity (aqueous leaf extract), leishmanicidal activity (aqueous and methanol extracts) [16]	In vitro (semi-automated microdilution assay, anti-leishmanial assay, anti-promastigote assay, anti-amastigote assay, MTT assay) [16]	The aqueous and methanol leaf extracts were reported to have low cytotoxicity against human embryonic lung fibroblast (HELF) cell lines (CC_50_ > 500 µg/mL) [16]
*Allium sativum* L.	Malaria, wound disinfectant, intestinal infections [17], cold [18], aphrodisiac [19]	Chemoprophylaxis against lead nitrate induced toxicity in mice [20], increase in the weight of seminal vesicles and epididymis of male animals and elevation of sperm count [21], antibacterial and antifungal activity (essential oil extracts) [22]	In vivo (Swiss albino mice) [20], in vivo (Swiss albino mice) [21], in vitro (disc diffusion and yeast glucose Chloramphenicol Agar method) [22]	The LD_50_ in rabbits was reported to be 3034 mg/kg with a maximum tolerated dose of 2200 mg/kg [23]. Mortality in rabbits was recorded at 3200 and 4200 mg/kg. Anorexia and paralysis were observed in rabbits at high doses [23] The aqueous extract at a 300 mg/kg dose was reported to have mild toxicity symptoms in *Wistar* rats, but doses of 600 mg/kg and 1200 mg/kg were reported to elevate biochemical parameters. No toxicity was reported up to a dose of 2500 mg/kg and LD_50_ was reported to be > 5000 mg/kg [24]
*Rhus natalensis* Bernh.	Diarrhea, influenza [25] Respiratory disorders, Malaria [26]	Antinociceptive activity (dichloromethane-methanol extract) [27], antibacterial activity (aqueous extract) [25]	In vivo (Swiss albino mice) [27], in vitro (Standard plate count method) [25]	3-(*Z*)-heptadec-14-enyl) benzene-1-ol isolated from the ethyl acetate root extract of *R. natalensis* was reported to be toxic in brine shrimp larvae (LC_50_ = 7.25 µg/mL), induced apoptosis, and caused cell cycle arrest [28]
*Steganotaenia araliacea* Hochst.	Skin diseases [29], tuberculosis [30]	Antibacterial activity (aqueous and methanol root extracts) [31], uterotonic activity in uterine strips of pregnant rats [32], diuretic activity (aqueous, methanol, and ethanol stem bark extracts) [33]	In vitro (Agar well diffusion method) [31], ex vivo (*Wistar* rats; organ bath) [32], in vivo (*Wistar* rats) [33]	The 80% ethanolic stem bark extract was reported to be cytotoxic against MDA-MB-231 (breast), PANC-1 (pancreas), and HT-29 (colon) cancer cell lines [34] Dibenzocyclo-Octadiene, a lignan constituent was reported to have antimitotic activity [35]. Steganacin (an isolated compound) was reported to inhibit the polymerization of tubulin and to slow the depolymerization of pre-formed microtubules in the sea urchin egg assay [36]
*Carissa edulis* (Forssk.) Vahl	Respiratory infections [37], chest pains [38, 39]	Anti-bacterial activity (*S. aureus*, *E. coli*) [40]	In vitro (Agar well diffusion method) [40]	No acute toxicity was observed in mice at oral therapeutic doses of up to 250 mg/kg [41]. The methanol root bark and the aqueous and methanol root extracts were reported to be cytotoxic to brine shrimp larvae (LC_50_ = 255.06 µg/mL, 260.34 µg/mL, and 186.71 µg/mL respectively) [42, 43]
*Artemisia annua* L.	Fever [18]	Antimicrobial activity [44] antioxidant activity [45], cytotoxicity [46–49]	In vitro (Agar well diffusion method) [44] In vitro (total phenolic content assay, total flavonoid content assay, Ferric reducing antioxidant power assay, Trolox equivalent antioxidant capacity assay) [45], in vitro (Resazurin assay, cytogenetic assay) [46–49]	The dichloromethane and methanol extracts were reported to be cytotoxic against *Trypanosoma brucei brucei* (TC221 cells) [50] Artemisinin and quercetagetin 6,7,3′,4′-tetramethyl ether were reported to be cytotoxic against P-388, A-549, HT-29, MCF-7, and KB tumor cells [47]. The ethanol extract was reported to be cytotoxic against Molt-4 human leukemia cells and normal leukocytes [48]. The methanol extract was reported to be cytotoxic and genotoxic against meristem cells of *Allium cepa* [49]
*Microglossa pyrifolia* (Lam.) Kuntze	Ovarian cysts [17], malaria [17, 51]	Antioxidant activity (leaf extracts) [52], cancer cell line cytotoxicity [53], antiplasmodial activity (dichloromethane leaf extract) [54]	In vitro (2,2-diphenyl picryl hydrazyl (DPPH) assay) [52], In vitro (Resazurin assay) [53], In vitro (lactate dehydrogenase assay) [54]	The organic leaf extract was reported to be cytotoxic against CCRF-CEM leukemia and decreased cell growth by 48% [53]
*Tithonia diversifolia* (Hemsl.) A. Gray	Diabetes, malaria [55, 56], abscesses, snake bite [56]	Antiplasmodial activity (ethanol leaf extracts) [57], antibacterial and antifungal activity (aqueous and ethanol leaf extracts) [58], antiplasmodial activity [59]	In vivo (Swiss albino mice) [57], in vitro (Agar diffusion method) [58], In vitro (Semi-automated microdilution technique) [59]	Sesquiterpenoids isolated form the 80% ethanol extract of aerial parts were reported to be cytotoxic against HL-60 leukemia cells [60] Acetyltagitinin E and Tagitinine-F (leaf isolated compounds) were reported to be selectively cytotoxic against Hep G2 human hepatocellular carcinoma cells [61]. Tagitinin C (isolated from the leaves) was reported to be cytotoxic against colon cancer, other malignant cell lines [62, 63], and brine shrimp larvae [64]
*Kigelia africana* (Lam.) Benth.	Pneumonia [65], tuberculosis [30], measles in children [39]	Antibacterial activity (ethanol stem bark and fruit extracts) [66], antifungal activity [67], antibacterial, antifungal, antigiardial, and anticancer properties (Aqueous and methanol fruit extracts) [68]	In vitro (Micro titre plate bioassay) [66], in vitro (Agar diffusion method) [67], in vitro (Modified disc diffusion method) [68]	A 2000 mg/kg oral dose of the aqueous extract of the fruit was reported to cause hepatorenal toxic effects in *Wistar* rats [69] An 80% methanol extract of the fruit and roots was reported to be cytotoxic to brine shrimp larvae (LC_50_ = 240 µg/mL and 7.2 µg/mL respectively) [70] The aqueous bark extract was reported to be toxic to the African catfish (*Clarias gariepinus*) [71] The aqueous fruit extract was reported to be toxic to * Artemia franciscana* nauplii toxic with an LC_50_ value of 477 µg/mL [68] Compounds isolated from the hexane fraction of the stem bark were reported to be toxic against LLC/MK2 (monkey kidney epithelial cells) [72] The aqueous stem bark extract had a dose-dependent mortality on culet mosquito larvae [73] The ethanol stem bark extract was reported to be nontoxic to brine shrimp larvae (LC_50_ > 1000 µg/mL) [74]
*Commiphora africana* (A.Rich.) Engl.	Malaria, fever [75], swollen testicles, and abdominal pains [39], pneumonia [25]	Antifungal and antibacterial activity (Ethanolic root extract) [76]	in vitro (Agar diffusion technique) [76]	The 95% ethanol extract was reported to be nontoxic in mice and no mortality was observed even at concentrations of up to 5000 mg/kg. However, drowsiness in doses between 1200 and 5000 mg/kg was reported [77] The compounds isolated from the methanol stem bark fraction (resveratrol derivatives) were reported to have low cytotoxicity on prostate cancer cell lines [78]. The ethanol root extract was reported to be nontoxic in brine shrimp larvae [74]
*Warburgia salutaris* (G.Bertol) Chiov	Chest complaints, cough, fever, pneumonia [79], yellow fever [80], common cold, malaria [81], Aspergillosis [82]	Fungicidal activity against Fusarium species (Acetone extract) [83] antimycobacterial activity against *S. aureus, B. subtilis, S. epidermis, M. luteus, E. coli,* and *K. pneumoniae*) [84]	in vitro (Hole plate diffusion method, microdilution method) [83], in vitro (Bioautography assay) [84]	The acetone leaf extract was reported to be cytotoxic against cancer cell lines [85]
*Carica papaya* L.	Malaria, liver disease [12], tuberculosis [30], malaria, [86, 87], fever [18]	Antibacterial activity (Methanol root extract) [88], antitumour activity and immunomodulatory effects (Aqueous leaf extract) [89]	in vitro (Cup plate agar diffusion method) [88], in vitro (Cell viability assay, caspase assay, microarray analysis) [89]	The aqueous and ethanol leaf extracts were reported to be cytotoxic on human oral squamous cell carcinoma SCC25 cell lines [90] The aqueous leaf extract was reported to disrupt cell division and to induce mitotic spindle disturbance in *Allium cepa* [91] The methanol leaf extract was reported to be cytotoxic against LLC-MK2 cell lines [92] The aqueous leaf extract was reported to be non-toxic in Sprague Dawley rats at a 2000 mg/kg dose [93] No morphological alterations were reported in Sprague Dawley rats treated with a 28-day repeated oral dose of 2000 mg/kg [94] Aqueous and ethanol leaf extracts were reported to be nontoxic at doses of up to 5000 mg/kg [95] The methanol leaf, root, and stem bark extracts were reported to be nontoxic against MRC-5 cell lines (CC_50_ > 32 µg/mL) [96]
*Terminalia brownii* Fresen	Cough, bronchitis [97, 98], allergy, diabetes, malaria [25, 98], clotting agent, coughs and joint stiffness [99]	Anti-fertility effect (Ethyl acetate extracts) [100], antibacterial activity against *S. aureus, E. coli*, and *B. subtilis* (Aqueous bark extract) [25]	in vivo (Swiss mice) [100], in vitro (Standard plate count method) [25]	Doses of between 500 and 1000 mg/kg of the methanol root bark extracts were reported to cause dullness and decreased activity of Swiss albino rats [101]
*Ipomoea kituiensis* Var	Constipation, digestive disorders [99]	Acaricidal activity (Methanol:DCM (1:1 v/v) leaf extract) [102]	in vivo (Modified larval packet test) [102]	The aqueous extract was reported to be moderately toxic to brine shrimp larvae (LC_50_ = 136.96 µg/mL) [102]
*Euclea divinorum* Hiern.	Stomachache [103], bleeding [104], diarrhea, typhoid , stroke [105], toothache [99]	Contractile activity of isolated rabbit uterine strips (aqueous and ethanol root bark extracts) [106]	ex vivo (Organ bath; Swiss white rabbits) [106]	The aqueous and organic root extracts were reported to cause retarded growth and altered biochemical parameters in mice [107] The methanol root extract was reported to be cytotoxic against MEC-5 fibroblast cells (IC_50_ = 27.5 ± 3.6 µg/mL) [108]
*Croton megalocarpus* Del.	Influenza, pneumonia, wounds, family planning, typhoid, over bleeding during menstruation cycle and birth [105]	Antibacterial and antifungal activities (petroleum ether and aqueous leaf extracts) [109], antifungal activity (The methanol leaf extract) [110]	in vitro (Agar well and disc diffusion assays) [109], in vitro (Agar well diffusion technique) [110]	The LC_50_ was reported to be < 250 µg/mL in the brine shrimp lethality assay [111]
*Croton dichogamous* Pax	Chest congestion (wheezing) [112] Polio like-symptoms, gonorrhea, chest pains [39] Threatened abortion, infertility [113] Pesticidal activity [114]	No reports	No reports	No reports
*Harungana madagascariensis* Lam. Ex Poir	Gastrointestinal disorders [115]	Antibacterial activity against *B. subtilis, S. aureus, E. coli, P. aeruginosa* (Aqueous leaf extract) [115], antibacterial activity against *S. typhi, S. paratyphi, S. paratyphi B* and *S. typhimurium* (Aqueous extracts) [116], antibacterial activity (Astilbin or 3-O-α-l-rhamnoside-5,73,4′-tetrahydroxydihydroflavonol) [117]	in vitro (Modified agar well diffusion method) [115] In vitro (Broth dilution technique) [116], in vitro (Solid dilution method, bioautography) [117]	The aqueous leaf extract was reported to induce liver damage at high doses of > 100 mg/kg and > 200 mg/kg in female and male rats respectively [118]. A 400 mg/kg dose of the iso saline leaf extract administered intraperitoneally in Sprague-Dawley rats significantly elevated serum levels of alanine and aspartate aminotransferase, and significantly lowered the blood glucose levels [119].
*Gladiolus dalenii* Van Geel	Epilepsy, diarrhea, nasopharyngeal infection, intestinal spams [120]	Antibacterial activity against *S. pyogenes, K. pneumoniae* (95% ethanolic extract) [121] Antifungal activity against *Aspergillus niger* (1:1 dichloromethane/methanol (1:1) extract) [122]	in vitro (Agar well diffusion method) [121], in vitro (Disc diffusion method) [122]	Reported to contain cytotoxic substances that affect mitotic active tissue [123]. There was no indication of mutagenesis when dichloromethane and 70% ethanol extracts were tested on *S. typhimurium* (Ames test) (TA98) [124]
*Clerodendrum myricoides* (Hochst.) R.Br.ex Vatke	Malaria [125] Febrile convulsions, Abdominal colic [126] Respiratory infections [37] Pneumonia [25]	Antibacterial and antifungal activity (Organic root extract) [127], antibacterial activity (Aqueous and methanol leaf extract) [128], antiplasmodial activity (Methanol leaf extract) [129]	in vitro (Agar disc diffusion method) [127], in vitro (agar diffusion method) [128], in vivo (Swiss albino mice) [129]	The dichloromethane root bark extract was reported to be nontoxic on L6 cells (IC_50_ > 90 µg/mL) [130]. The methanol root extract was reported to be toxic to brine shrimp [131]
*Plectranthus barbatus* Andr.	Abdominal pain, diarrhea [132], tuberculosis [30], malaria [133], wounds, swelling, joint pain, stomach problems, malaria [134], ashtma [135]	Larvicidal properties (Eugenol, α-pinene and β-caryophyllene l) [136], anticonvulsant activity (Hydroalcoholic leaf extract) [137], inhibition of HIV-1 enzymes, antioxidant and anti-inflammatory activities (Ethanol leaf extract) [138]	in vivo (Third instar mosquito larvae) [136], in vivo (Swiss albino mice) [137], in vitro (MTT assay, flow cytometric analysis, HIV-1 protease fluorogenic assay, HIV-1 transcriptase colorimetric assay, DPPH free radical scavenging assay) [138]	The ethanol extract was reported to have low cytotoxicity against PBMCs and TZM-bl cell lines (IC_50_ values = 83.7 and 50.4 µg/mL respectively) [138] The methanol leaf extract was reported to be toxic to *Artemia salina* (LC_50_ = 186.33 µg/mL) [139] The chloroform aerial part extract was reported to reduce the viability of undifferentiated/anaplastic thyroid cancer cell lines [140]
*Vitex doniana* Sweet	Hypertension, diabetes, ulcers [141], malaria, measles [142], gastroenteritis, diarrhea [143], diuretic, diabetes [144]	Antimicrobial activity (Methanol stem bark extract) [145, 146], antioxidant activity (Aqueous leaf extract) [147], wound healing properties (Hydroalcoholic stem bark extract) [148]	in vitro (Paper disc assay method, Agar well diffusion method) [145, 146], in vitro (DPPH assay) and in vivo (Swiss albino mice) [147], in vivo (ICR mice) [148]	The organic leaf and bark extracts were reported to be non-toxic to mammalian L6 cell lines (IC_50_ > 90 µg/mL) [149]
*Acacia robusta* Burch.	Malaria [150], fibroids [113]	Antifungal activity (Methanol root bark extract) [151]	in vitro (Broth dilution) [151]	The methanol stem bark extract was reported to be toxic to brine shrimp (LC_50_ = 108.5 µg/mL) [70]
*Albizia zygia* (DC.) J.J.Macbr.	Antimalarial activity [152, 153], anticancer [154], cough, fever, aphrodisiac, counter female sterility [155], bronchial disease, fever [156]	Antimicrobial activity (Methanol and hexane extracts) [155], anti-inflammatory and antioxidant activity (Ethanol stem bark extract) [157]	in vitro (Agar diffusion) [155], in vivo (chicks), and in vitro (DPPH) [157]	The ethanol stem bark extract was reported to be nontoxic against MRC-5 cells (> 64 µg/mL) [96] The methanol extract was reported to be more toxic to brine shrimp than the non-polar n-hexane extract (LC_50_ 1.70 µg/mL compared to 174.19 µg/mL) [155]
*Rhynchosia elegans* var. elegans	Malaria, common cold, fever [12]	No reports	No reports	No reports
*Tamarindus indica* L.	Malaria [158, 159], constipation, jaundice [97], aphrodisiac [19], general wellbeing [18], sexually transmitted infections [99]	Antibacterial activity against *P. mirabilis* (Acetone stem bark extract) [160], antibacterial activity against *S. aureus, E. coli,* and *P.aeurigenosa* (Aqueous pulp extract) [161]	in vitro (Paper disc diffusion method) [160], in vitro (disc diffusion method) [161]	The LD_50_ values of various crude extracts and 25–50% fractions were reported to be in the range of between 832 and 5019 µg/mL [162] The acute oral toxicity studies of the pulp extract at 3000 mg/kg and 5000 mg/kg body weight resulted in no mortality in *Wistar* albino rats [163]
*Tylosema fassoglense* (Kotschy ex Schweinf.) Torre & Hillc.	Epilepsy, infertility in women, renal disease, cancer [132]	Antibacterial activity (Methanol extracts) [164], antifungal activity, and cytotoxicity (Ethyl acetate extracts) [165]	in vitro (disk-diffusion assay) [164], in vitro (Broth microdilution method) and in vivo (brine shrimp cytotoxicity) [165]	The dichloromethane, ethyl acetate, and aqueous extracts were reported to be toxic to brine shrimp (LC_50_ = 203.66 µg/mL, 7.58 µg/mL, and 17.57 µg/mL respectively) [165]
*Azadirachta indica* (L) Burm.	Malaria [159, 166], scabies, control blood sugar levels [167], tuberculosis [30]	Antibacterial activity against *S. typhi* and antifungal activity against *C. albicans* (n-hexane extract) [168], antioxidant and antibacterial properties (50% ethanol leaf extract) [169]	in vitro (Ditch well diffusion method) [168], in vitro (Agar well diffusion method [169]	The aqueous and methanol leaf extracts were reported to be non-toxic against MRC-5 cells (CC_50_ > 32 µg/mL) [96] The methanol leaf extract was reported to be toxic to brine shrimp larvae (LC_50_ = 233.061 µg/mL) [42] The aqueous and methanol leaf extracts were reported to be toxic to brine shrimp larvae (LC_50_ = 101.26 and 61.43 µg/mL respectively) [43].
*Khaya senegalensis* Desr. A. Juss	Diabetes, hypertension [170], hepatic inflammations, sinusitis [97]. malaria [87]	Antibacterial activity against *S. enterica* subsp. *Enterica serovar typhi* (50% ethanolic leaf extract) [171], in vivo hypoglycemic activity (Ethyl acetate extract) [172], hepatoprotective effects [173], antioxidant activity (Ethanolic extract) [174]	in vitro (Agar well diffusion method) [171], in vivo (rats) [172], in vivo (rats) [173], in vitro (DPPH radical scavenging assay, deoxyribose assay, Nitric oxide radical scavenging assay) [174]	Orally administered ethanol stem bark extract in rats at a dose of 2 mg/kg for 18 days was reported to induce the synthesis of liver enzymes [175]. The subchronic administration of the aqueous stem bark extract to rats was reported to affect the cellular integrity of vital organs of the body [176]. Sub-chronic administration of the aqueous stem bark extract in albino rats was reported to cause the elevation of liver enzymes, and to Increase plasma total protein, blood urea, and creatinine [177].
*Mollugo nudicaulis* Lam.	Whooping cough and jaundice [178]	Antioxidant and antibacterial activity(Methanol leaf extract) [179], antidiabetic properties (Ethanolic whole-plant extract) [180]	in vitro (Total phenolic content assay, total flavonoid content assay, ABTS scavenging activity assay, DPPH radical scavenging assay, agar disc diffusion assay) [179] in vivo (*Wistar* rats) [180]	No reports
*Moringa oleifera* Lam.	Malnutrition [75], tuberculosis [30], loss of memory, prostate cancer [105], flu, asthma, hypertension, malaria [181]	Antibacterial activity against *P. aeruginosa* and *S. aureus* (Fresh leaf juice and aqueous seed extracts) [182], chemoprophylaxis against Artesunate-amodiaquine induced liver damage (aqueous-methanol leaf extracts) [183]	in vitro (Paper disc diffusion method) [182], in vivo (*Wistar* rats) [183]	The aqueous leaf extract was reported to increase the cytotoxic effect of chemotherapy on pancreatic cancer cells [184] The organic leaf extract was reported to be toxic to brine shrimp larvae [185] The aqueous extract was reported to be strongly cytotoxic on Hela cells [186]
*Eucalyptus camaldulensis* Dehnh	Tuberculosis [30], malaria, liver disorders [75], respiratory tract congestion, chronic bronchitis, coughing, tuberculosis [187]	Antibacterial activity (Essential oil from the leaves) [188], antibacterial activity against *H. pylori* (N-hexane and chloroform leaf extract) [189], antimycobacterial activity against *M. tuberculosis* and *M. bovis* strains (Methanol extracts) [190]	in vitro (Aromatogram, micro atmosphere test, broth dilution method [188], in vitro (Agar disc diffusion) [189], in vitro (Resazurin microtiter assay) [190]	The aqueous-acetone extract was reported to be cytotoxic on MCF-7 and HCT-116 cell lines [191] The essential oils from fresh leaves were reported to inhibit egg hatchability and to suppress the second stage juvenile viability of root-knot nematode *Meloidogyne incognita* [192] The methanol leaf extract was reported to be cytotoxic against human breast cancer cell lines (MCF 7 and MDA-MB-231) cell lines [193] The methanol leaf extract was reported to be cytotoxic on P19 embryonal carcinoma cells [194]
*Syzygium cumini* (L.) Skeels.	Asthma, bronchitis, sore throat [195], coughing, diabetes, dysentery, ringworms, inflammation [196], diarrhea, dysentery, wounds, constipation [167]	Anti-inflammatory activity in mice (Ethanol bark extract) [197], hypoglycemic activity (Aqueous bark extract) [198]	in vivo (mice) [197] in vivo (rats) [198]	The methanol extract was reported to have an LD_50_ value of > 5000 mg/kg in mice [199] The ethanol extract was reported to be nontoxic to rats at doses of up to 5000 mg/kg [200] The ethanol bark extract was reported to be nontoxic in mice at doses of up to 10.125 g/kg [197]
*Ximenia americana* L.	Throat infection, amenorrhea, wound healing, pain [201]	Antimicrobial activity against *E. coli, P. aeruginosa, and P. vulgaris* (bark, leaf, and root extracts) [202], antioxidant activity (Methanol stem bark extract) [203]	in vitro (cup-plate agar diffusion method) [202], in vitro (DPPH radical scavenging assay) [203]	The methanol stem bark extract was reported to be nontoxic against MRC-5 cell lines (CC_50_ = 64 µg/mL) [96]
*Clematis hirsuta* Guill. & Perr	Colds, cleanser [105], chest problems [134]	Antifungal activity against *C. albicans* [204]	in vitro (Liquid dilution method) [204]	The oral administration of an 80% methanol leaf extract did not result in any physical signs e.g. depression, decrease in feeding activity, and hair erection in Swiss albino mice [205]
*Gardenia ternifolia* Schumach. & Thonn.	Hypertension [170] Treat dysentery, urinary tract infections [206]	Antimicrobial activity against *C. coli, C. jejuni, S. aureus* (Aqueous extract) [206], antiplasmodial activity (80% methanol root bark extract) [207], viricidal activity against African Swine Fever Virus (Ethanol root extract) [208]	in vitro (disc diffusion method) [206], in vivo (Swiss albino mice) [207], in vitro (Plaque titration technique) [208]	The ethanol root extract was reported to be non-toxic on human carcinoma cell lines [209]
*Keetia gueinzii* (Sond.) Bridson	Malaria [166]	Antimycobacterial activity against pathogenic and non-pathogenic *Mycobacterium* species [210]	in vitro (Bioautography and the modified two-fold serial dilution microplate method; anti mycobacterial activity) [210], in vitro cytotoxicity; MTT assay [210]	The acetone leaf extract was reported to have an LC_50_ of 0.142 in vero cell lines and 0.063 in SI C3 A cell lines [210]
*Harrisonia abyssinica* Oliv.	Arthritis, sexually transmitted infections [26], stomach ache, coughs, malaria [99] Malaria [133]	Antifungal activity [211], antiviral, antifungal, antibacterial, and molluscicidal activity [212]	in vitro (Agar well diffusion method) [211], in vivo (Molluscs) [212]	The methanol root bark extract was reported to be cytotoxic in brine shrimp (LC_50_ = 198.498 µg/mL) [42]
*Teclea nobilis* Del.	Antipyretic [213], malaria, headache, joint pains, common cold, pneumonia, intestinal worms, chest pain [134], arthritis [39]	Antipyretic and analgesic activity and found to be weakly active against carrageenan edema (Ethanol leaf extract) [214], anti-inflammatory, analgesic, and antipyretic activities (Acetonitrile leaf extract, hexane leaf extract, and Lupeol) [215], anti-caseinolytic activity against *B. arietans* venom (Methanol root extract) [216]	in vivo (*Wistar*-Nossan rats) [214], in vivo (*Wistar* rats) [215], in vitro (Spectrophotometry) [216]	The dichloromethane and ethanol extracts of aerial parts were reported to be cytotoxic to brine shrimp (LC_50_ = 75.5 µg/mL and 156.6 µg/mL respectively) [217]
*Toddalia asiatica* L.	Sore throat, Malaria [218], fever, stomach ache [219], abdominal pains, gynecologic disorders including infertility, common colds, cancer, renal disorders [132], tuberculosis, [30], common cold, fever, malaria, pneumonia, chest pain [134], colds, respiratory diseases e.g. cold, asthma, chest pain, toothache [105], malaria and bark for respiratory disorders [39]	Larvicidal activity (Hexane, acetone, and methanol leaf extracts) [220], antifungal activity against *Candida albicans* (Ethyl acetate leaf extracts) [221], antinociceptive and anti-inflammatory effects (1:1 dichloromethane-methanol root extract) [222]	in vivo (*Aedes egyptii* and *Culex quinquefasciatus*) [220], in vitro (Agar well diffusion method) [221], in vivo (Swiss albino mice) [222]	Compound **13** isolated from the root was reported to be cytotoxic against the MCF-7 cell line (IC_50_ = 8.7 µg/mL) but was inactive on Vero cells. Alkaloid **11** was reported to be cytotoxic against KB, NCI-H187, MCF-7, and vero cell lines (IC50 values ranging from 0.8 to 11.6 µg/mL) [223] Essential oils from the leaves were reported to be cytotoxic against breast (MCF-7) and colorectal (HT-29) cancer cell lines [224] (IC_50_ values = 7.80 µg/mL and 100.0 µg/mL respectively). Benzo[*c*]phenanthridine and secobenzo[*c*]phenantridine alkaloids isolated from the ethanol root extract was reported to be cytotoxic on tumor cell lines [225] The acute toxicity and cytotoxicity of the aqueous, ethyl acetate, and methanol leaf extract and root extracts were reported to be > 1000 mg/kg (LD_50_) and > 100 µg/mL (CC_50_) respectively [219] The alkaloid (1,3)benzodioxolo(5,6-c)phenanthridine, 12,13-dihydro-2,3-dimethoxy-12-methyl-(dihydronitidine) was reported to be highly cytotoxic to human lung adenocarcinoma (A549) cells [226]
*Zanthoxylum chalybeum* (Eng) Engl.	Tuberculosis [30], malaria [166], pneumonia, [134], cough, cervical cancer [227]	Antibacterial activity against *S. aureus* (Methanol extracts) [128], antihyperglycemic activity (Aqueous stem bark extract) [228], antimicrobial activity against *B. cereus* and MRSA (Aqueous root bark extract) [229], antiplasmodial activity (Aqueous root bark extract) [230]	in vitro (Agar well diffusion method) [128], in vivo (*Wistar* rats) [228], in vitro (Agar well diffusion method) [229], in vivo (Swiss albino mice) [230]	The methanol root bark extract was reported to be toxic to brine shrimp (LC_50_ = 68.9 µg/mL) [70] The ethanol root extract was reported to be toxic in brine shrimp larvae (38.51 µg/mL) [74] The organic root extract of *Zanthoxylum chalybeum (Eng) Engl. (Rutaceae)* was reported to be cytotoxic in brine shrimp (LC_50_ = 11 µg/mL) [231] A 2000 mg/kg dose of the aqueous and organic extracts were reported to be nontoxic in mice [230] The organic extract was reported to be toxic in brine shrimp larvae (LC_50_ = 42.73 µg/mL) [230]
*Zanthoxylum gilletii* (De Wild.) P.G.Waterman	Malaria [51]	Antiplasmodial activity against *P. falciparum* (50% MeOH in CH_2_Cl_2_ extract) [232]	in vitro (non-radioactive Malaria SYBR Green I assay) [232]	Lupeol (an isolated compound) was reported to be cytotoxic against a panel of drug-sensitive and MDR tumor cells via multiple mechanisms with marginal or no effect on normal cells at similar doses [233]. The ethanol stem bark extract was reported to be cytotoxic on leukemia CCRF-CEM cells (IC_50_=9.04 µg/mL) [234].
*Cissus rotundifolia* (Forssk.) Vahl	Threatened abortion/contraception [113] Pain [128] Malaria, liver disease and otitis [235] Malaria [159]	Antibacterial activity (Buffered methanol (80% methanol and 20% PBS) and acetone) [236], hypoglycemic activity(Aqueous leaf extracts) [237]	in vitro (Agar well disc diffusion assay) [236], in vivo (*Wistar* rats) [237]	The methanol (70%) extract of aerial parts was reported to be more cytotoxic on MCF-7 (breast cancer) cell lines than doxorubicin (IC50 = 0.77 µg/mL and 3.45 µg/mL respectively) [238]
*Rhoicissus revoilii* Planch	Pneumonia, tonsillitis [239]	Antifungal activity against *C. albicans* (Ethanol extract) [239]	in vitro (Agar well disc diffusion assay) [239]	No reports

## Discussion

### Socio-demographic information of herbalists in the study area

Many of the herbalists interviewed in this study were older members of the society. It has previously been reported that traditional herbal practice is usually a preserve of the older members of the society [240, 241]. It is also important to note that it is often harder for the younger generation of herbalists to be accepted by their communities as they are considered to be inexperienced in key tenets of traditional herbal medicine [240, 241]. The observation that many of the interviewed herbalists had not received any formal education seems to agree with what has been observed by other authors [241].

### Diversity of medicinal plants identified in the study area and their use

The Leguminosae plant family was the most dominant family indicated for respiratory illnesses in the study area. According to Christenhusz and colleagues, Leguminosae has a large global distribution and is the 3rd largest plant family in the world (after Orchidae and Asteraceae) [242]. The worldwide distribution of this plant family may have some influence on the decision of herbalists to use the plants from this family [243].

The predominance of trees as a source of herbal therapies may have something to do with their abundance, easy availability throughout the year, and resistance to drought and seasonal variations [243–245]. Leaves are considered by herbalists to be important photosynthetic organs [241, 243]. Thus, it is not surprising that they were the most frequently used plant parts in the study area.

It was disturbing to note that many of the herbalists in the area were uprooting the plants that they used for making some of the indigenous remedies. Furthermore, in the course of the interview, some of the herbalists had reported that *Warburgia salutaris* and *Zanthoxylum gilletii* were no longer available in some parts of Kisumu East Sub County owing to poor conservation practices. According to Maroyi, it is not advisable to over use the roots and stem barks of plants for medicinal value as this may sabotage plant conservation efforts [246]. Notwithstanding, some herbalists reported that they only collected plant parts in quantities that were enough for their work and which would not hamper conservation efforts. It is also worth mentioning that a local name for *Acanthus polystachyus* was not available. Instead, there was a consensus among the interviewed herbalists that ‘Nyanandi’ was the closest semblance to a name that this plant could be given on account of the assertion that it may have originally have been brought in from Nandi County which happens to be an immediate neighbor of Kisumu County.

### Dosage, mode of preparation, and route of administration

Teaspoons and tablespoons were used for measuring the dosages of powdered plant materials such as barks, stems, or roots while glasses or cups were used for measuring doses of concoctions or decoctions. While the use of 300/500 mL cups was commonly recommended by the herbalists as a means of measuring the dosages of concoctions/decoctions to be used, there was ambiguity in how this was applied. This trend was also observed in a previous report where medicinal plants used for maternal healthcare in Katsina state, Nigeria were surveyed [18].

Decoctions and concoctions were the most common method of preparing indigenous remedies and was done by the herbalist or by the patient who was given instructions on how to make the preparation. The process often involved harvesting the plants, drying them in the sun or in the house for a period of several days, and crushing them into powder with the aid of a homemade mortar and pestle.

The preparations would then be stored in plastic soda bottles that varied between 500 mL and 2 L and sold to the patients directly or in the market. Powdered plant parts could be included in tea and administered orally.

The route of administration was majorly orally. In the case of *Eucalyptus camaldulensis*, decoctions were prepared by boiling the leaves in an earthen pot and the patient was advised to cover themselves with a blanket such that the emanating steam completely engulfed them. This was done over a period of time and the patient would later be advised to take 2 teaspoons of the decoction in the event that they had a common cold. Patients were asked to revert back to the herbalist for further directions in case they did not feel better. It is worth noting that many of the interviewed herbalists were of the opinion that their remedies rarely failed. In the minds of the herbalists, the failure of the remedies to work was largely due to the incapacity of the patients to follow the instructions issued by the herbalists.

The interviewed herbalists were of the opinion that their remedies had minimal side effects. However, it is not clear whether these herbalists had the capacity to identify any adverse events or whether they had any mechanisms to report such cases whenever they occurred.

### Pharmacological reports and toxicology of the medicinal plants documented in this study

To the best of our knowledge, this is the first study to document the medicinal plants used in the management of respiratory illnesses by herbalists in Kisumu East Sub County. It is interesting to note that up to 84.1% of the medicinal plants documented in this study have previously been reported to be effective against *Staphylococcus aureus, Escherichia coli, Pseudomonas aeruginosa, Aspergillus* spp, and *Candida albicans*. These microorganisms have been associated with pneumonia and tonsillopharyngitis [247].

The most cited plants in this study were *Warburgia salutaris, Zanthoxylum gilletii, Carissa edulis, Tylosema fassoglensis*, and *Harrisonia abyssinica. Carissa edulis* and *Clerodendrum myricoides* have been reported to be useful in the management of asthma, cough, and cold [37, 105]. The similarity of our observations to those made by previous authors seems to suggest that there may in fact be a consensus among herbalists from different communities with regard to the usefulness of some of the medicinal plants in their environment.

Toxicological data was not available for 4 species of plants including *Croton dichogamous, Rhynchosia elegans, Mollugo nudicaulis,* and *Rhoicissus revoilii.* Moreover, there was no pharmacological data on *Croton dichogamus,* and *Rhynchosia elegans*. This may be a potential gap that may need filling in the future.

## Conclusions

The predominant use of roots, root barks, and root tubers in preparing decoctions by herbalists in the study area threatens the ecological survival of some of the plant species used. The preservation of ethno medicinal knowledge in the study area is a pressing concern considering the advanced age and little formal education of the herbalists interviewed. Plans to conserve some of the medicinal plants documented in this study should be initiated. There is a need to scientifically scrutinize the medicinal claims made by the herbalists interviewed in this study.

## Limitations

The dosage frequency, duration of treatment, and storage condition of the powdered plant material, decoctions, or concoctions were not captured during the interviews. Information on the duration of treatment was also not captured.

## Supplementary information

**Additional file 1.** Summary of the questionnaire used to interview herbalists in Kisumu East Sub County.

## Data Availability

All data generated or analyzed during this study are included in the text.

## Abbreviations

LRTI: Lower respiratory tract infections
COPD: Chronic obstructive pulmonary disease
WHO: World Health Organization
BAUEC: Biosafety animal use and ethics committee
RFC: Relative frequency of citation
