# Supplementary material for: Medicinal plants used in managing diseases of the respiratory system among the Luo community: an appraisal of Kisumu East Sub-County, Kenya
Source: Chin Med. 2020 Sep 3;15:95. doi: 10.1186/s13020-020-00374-2 (PMC7469313; doi:10.1186/s13020-020-00374-2)
Supplement: Supplementary file 1 — Additional file 1. Summary of the questionnaire used to interview herbalists in Kisumu East Sub County. [file 13020_2020_374_MOESM1_ESM.docx]

**Questionnaire to collect data on the socio-demographic information of traditional medicine practitioners in Kisumu East Sub-County and the medicinal plants used in managing diseases of the respiratory system**

Dear respondent

Below is a questionnaire that seeks information on your expertise in ‘Medicinal plants used in managing diseases of the respiratory system in Kisumu East Sub County’. I would be grateful if you would be willing to be interviewed to facilitate the completion of the questionnaire as best and as honestly as you can. The information you give will assist me in documenting the various plants used in preparing indigenous remedies used in managing infections of the respiratory system in Kisumu East Sub County. Any information provided by you will be treated with the utmost confidentiality and no single response will be reported on its own, but as a summation of all the responses. I am requesting 20 minutes of your time to conduct the interview.

Thanking you for your time.

James Kiamba Mailu

Participant file Number………………..

1. **Participant Socio-demographic information**
2. Gender Male Female
3. Age
4. Level of education

No formal education Primary education

Post-secondary education Secondary education

1. Ward/Village
2. **Information on the practice of herbal medicine**
3. Have you heard of traditional medicine where medicinal plants are used? Yes

No

1. Do you practice traditional herbal medicine? Yes No
2. If yes, for how many years have you been practicing? .......................................
3. **Information on the diseases treated, and methods used in the treatment**
4. What are the most common illnesses that you treat using herbal medicine in this area? ………………………………………………

………………………………………………

………………………………………………

………………………………………………

………………………………………………

1. How do you treat some of the common illnesses in this area? .......................................................................

.......................................................................

.......................................................................

.......................................................................

1. **Information on the medicinal plants used in the treatment**
2. When medicinal plants are used for treatment, which ones do you use in your practice? ....................................................................

....................................................................

....................................................................

....................................................................

....................................................................

....................................................................

1. How do you prioritize the use of these plants in the management of respiratory illness?

……………………………………………

……………………………………………

……………………………………………

……………………………………………

……………………………………………

……………………………………………

1. What are the local names of the medicinal plants that you use in your practice? …………………………………………….

…………………………………………….

…………………………………………….

…………………………………………….

…………………………………………….

1. Which parts of the medicinal plants do you use to treat respiratory illnesses? ……………………………………………

……………………………………………

……………………………………………

……………………………………………

……………………………………………

……………………………………………

1. How do you prepare these medicinal plants using the parts you have stated above? ……………………………………………

……………………………………………

…………………………………………....

…………………………………………….

…………………………………………….

…………………………………………….

1. How is the prepared medicinal plant part administered and for how long? ………………………………………………

………………………………………………

………………………………………………

………………………………………………

………………………………………………

………………………………………………

1. In what form are the preparations used?

........................................................................

1. How do you store the plant for future use? ………………………………………………

………………………………………………

……………………………………………….

…………………………………………….…

……………………………………………….

1. What side effects have you observed in your patients who use these medicinal plants? ………………………………………………

………………………………………………

………………………………………………

………………………………………………

………………………………………………
